# Scalable networks of wireless bioelectronics using magnetoelectrics

**DOI:** 10.21203/rs.3.rs-5005441/v1

**Published:** 2024-09-24

**Authors:** Joshua E. Woods, Fatima Alrashdan, Ellie C. Chen, Wendy Tan, Mathews John, Lukas Jaworski, Drew Bernard, Allison Post, Angel Moctezuma-Ramirez, Abdelmotagaly Elgalad, Alexander G. Steele, Sean M. Barber, Philip J. Horner, Amir H. Faraji, Dimitry G. Sayenko, Mehdi Razavi, Jacob T. Robinson

**Affiliations:** 1 Department of Electrical and Computer Engineering, Rice University, Houston, TX, USA; 2 Texas Heart Institute, Houston, TX, USA; 3 Department of Neurosurgery, Houston Methodist, Houston, TX, USA; 4 Houston Methodist Research Institute, Houston, TX, USA; 5 Department of Neuroregeneration, Houston Methodist, Houston, TX, USA; 6 Department of Medicine, Cardiology, Baylor College of Medicine, Houston, TX, USA; 7 Department of Bioengineering, Rice University, Houston, TX, USA; 8 Applied Physics Program, Rice University, Houston, TX, USA; 9 Department of Neuroscience, Baylor College of Medicine, Houston, TX, USA

## Abstract

Networks of miniature bioelectronic implants would enable precise measurement and manipulation of the complex and distributed physiological systems in the body. For example, sensing and stimulation nodes throughout the heart, brain, or peripheral nervous system would more accurately track and treat disease or support prosthetic technologies with many degrees of freedom. A main challenge to creating this type of in-body bioelectronic network is the fact that wireless power and data transfer are often inefficient when communicating through biological tissues. This challenge is typically compounded as one increases the number of implants within the network. Here, we show that magnetoelectric wireless data and power transfer enable a network of millimeter-sized bioelectronic implants where the power transfer efficiency of the system improves as the number of implanted devices increases. Using this property, we demonstrate networks of wireless battery-free bioelectronics ranging from 1 to 6 implants where the wireless power transfer efficiency for the system increases from 0.2% to 1.3%, with each node in the network receiving 2.2 mW at a distance of 1 cm. We use this system for efficient and robust wireless data and power transfer to demonstrate proof-of-concept networks of miniature spinal cord stimulators and cardiac pacing devices in large animals. The scalability of this network architecture enabled by magnetoelectric wireless power transfer provides a platform for building wireless closed-loop networks of bioelectronic implants for next-generation electronic medicine.

## Introduction

Electrical neuromodulation treatments can often be improved by increasing the number of stimulation and recording sites within the body and projecting these sites to specific anatomical targets, which may differ for each patient. However, creating a personalized bioelectronic network is often limited by the number of contacts that can be wired to a central controller often referred to as an implanted pattern generator (IPG). FDA-approved bioelectronic devices only support up to four wires per implanted IPG. Alternatively, a distributed wireless network of bioelectronic devices would enable dozens of implants without the need to route wires through the body to each stimulation and recording site. Innovative wireless power transfer (WPT) technologies have recently enabled miniature bioelectronic implants based on RF ^1^, inductive coupling ^2^, volume conduction ^3,4^, ultrasound ^5^, and light ^6^. Despite such breakthroughs, these technologies are rarely tested in a large animal model where the implantation depth and distance between nodes would closely mimic clinical applications. The main challenge is that most WPT methods for bioelectronics struggle to power many implants simultaneously *and* deliver this energy over the specific areas and depths required for clinical neuromodulation. RF WPT can be used to create large networks of neural stimulation and recording devices in rodents. However, in miniaturized devices, these networks have only been demonstrated at distances of a few millimeters, which is insufficient to power devices implanted in large animals or human subjects ^1,2^. Nearfield inductive coupling struggles to power miniaturized devices because the power transfer efficiency falls off with the square of the area of the receiver, and there are tight alignment requirements ^7^. With ultrasound, light, and midfield WPT, energy must be focused on each node, making it difficult to scale to multiple implants because the focused energy must be divided between each implant ^5,8^, or power repeaters must be used ^9^.

Magnetoelectric (ME) WPT has unique properties that suggest it could support a scalable bioelectronic network. Firstly, ME can deliver very high power densities in excess of 2 mW/mm^2^ ^10^ to millimeter-sized bioelectric implants. This is because, unlike magnetic induction, the power transfer efficiency for ME scales with the area of the receiver (not the area squared) ^11^. Secondly, unlike ultrasound, light, and midfield technologies, ME WPT does not require energy to be focused on each receiver. This feature enables translational misalignment tolerances of several centimeters (comparable to the diameter of the transmitter) and angular misalignment tolerance of nearly +/− 90 degrees^12^. Finally, these implants are expected to place little load on the transmitter. Together, these features imply that as long as implants are placed within the transmitter’s field, one can add multiple devices at different positions, angles, and depths, and power them simultaneously without any additional power required from the transmitter. To test this hypothesis, we characterized the power transfer efficiency as we increased the number of ME receiving elements, built a network of 12 wirelessly-powered battery-free devices powered and controlled by a single transmitter, and demonstrated networks of digitally programmable bioelectronic implants in large animals.

## A wireless network of ME-powered devices

**Fig. 1 |:**
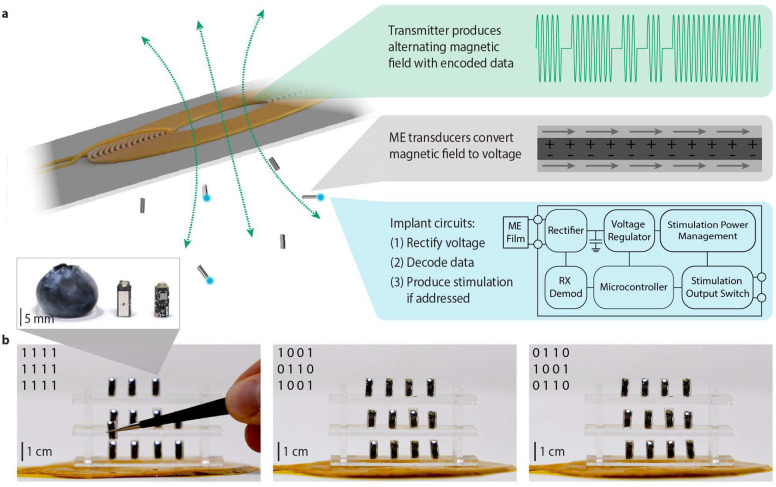
A network of wireless, battery-free, ME-powered devices. **a,** System concept of a single transmitter powering 6 ME nodes. **b,** A network of 12 simultaneously powered and individually addressable ME nodes shows examples of the digital programmability of the network, where each image shows a different programmed setting represented by the bit matrix in the upper left corner with 1 = on, 0 = off. (inset) Detailed view of an ME node next to a blueberry for scale.

To study the properties of ME-powered bioelectronic networks, we designed a system consisting of one alternating magnetic field transmitter and many mm-scale receivers (Fig. 1a). The ME materials we use to receive power and data are tri-layer laminates consisting of one 267 μm thick piezoelectric (PZT) layer sandwiched between two layers of 25 μm thick magnetostrictive Metglas layers. Using custom laser micromachining we cut these laminates to a 7.5 mm by 3 mm rectangle, which has a resonant frequency of 220 kHz. Power transfer occurs when the alternating magnetic field produces strain in the magnetostrictive layer, which is transferred to the bonded piezoelectric layer that converts this strain into an electrical potential. This power transfer is optimized when a static magnetic field biases the material at the inflection point of the strain curve, and the applied magnetic field matches the acoustic resonant frequency of ME film ^13^. During the power-transfer phase, the transmitter produces an alternating magnetic field at the acoustic resonant frequency of the mm-scale receivers. To transmit data, we use an on-off keying scheme consisting of 250 μs duration bits where the field either turns off, resulting in a dip in received voltage (a ‘0’), or remains on (a ‘1’). The ME material transduces this modulated alternating magnetic field into an alternating voltage, which is used to power a custom circuit that rectifies the voltage, decodes the data in the powering field, and produces the programmed output.

The carrier frequency on the order of hundreds of kilohertz provides advantages for bioelectronic data and power transfer; alternating magnetic fields in this frequency range show little absorption in biological tissue ^14^. This fact allows us to perform much of our benchtop testing in air, knowing that the results will translate well to biological tissue or liquids as previously characterized ^10,12^. The low absorption of magnetic fields at these frequencies also allows for safe exposure to magnetic fields up to 8 mT RMS according to the IEEE safety limits ^15^. All the studies performed here use magnetic fields within this safety limit.

To individually control each implant in a network that is simultaneously powered by the same transmitter, we designed each data payload to contain a device ID and output configuration settings. Each device receives every data payload, but only the device whose digital ID matches the ID in the payload will update its settings, while the other devices continue to operate as previously programmed. As an example, we show a network of 12 devices with LEDs that allow us to visualize the timing of the programmed stimulation (Fig. 1b). Each device contains one 7.5 mm by 3 mm ME film and a custom PCB that routes the electrical stimulation pulse to an LED for visualization. We broadcast 6-bit data payloads from the transmitter with a data rate of 4 kHz. These payloads contain a start and stop bit, a 4-bit ID, one bit to enable blinking, and one bit to trigger blinking on all currently enabled devices. Using this communication strategy, we can program different spatiotemporal stimulation patterns and visualize these patterns with a camera. (Fig. 1b, Supplementary Movie 1). Because the magnetic field is generated in a volume around the transmitter, and the devices have excellent misalignment tolerance, devices can be arranged in various configurations and orientations (Fig. 1b, Supplementary Movie 1).

## Power transfer efficiency increases with the number of receivers

**Fig. 2 |:**
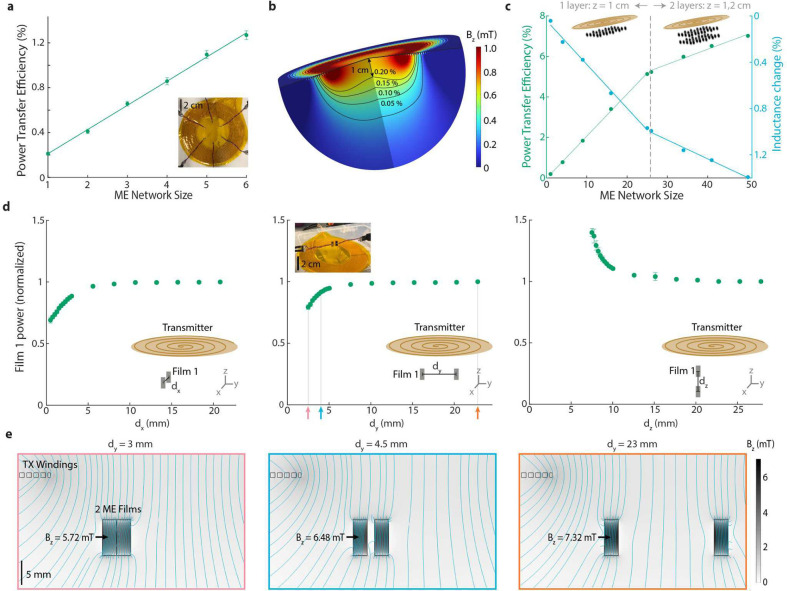
Scaling limits for ME networks. **a,** Experimental results show ME power transfer efficiency increases linearly with network size. ME films are placed symmetrically with the bottom 1 cm below the TX coil. Fit line: *0.22x - 0.004518, R*^*2*^ *= 0.9983,* n = 3 measurements of the same samples for each data point, error bars represent standard deviation. (inset) top view image of six ME films placed below the transmit coil. **e,** COMSOL simulation shows volumes within which the efficiency is greater than 0.20% (~34 cm^3^), 0.15% (~45 cm^3^), 0.10% (~61 cm^3^), and 0.05% (~93 cm^3^). **c,** Simulated network efficiency and inductance change as the number of ME films below the coil increases from 1 to 50 devices. Left efficiency fit line: *0.2081x − 0.02131, R*^*2*^ *= 0.9992.* Right efficiency fit line: *0.07429x + 3.382, R*^*2*^ *= 0.9846.* Left inductance fit line: *0.03782x + 0.04082, R*^*2*^ *= 0.9941.* Right inductance fit line: *0.01628x + 0.5837, R*^*2*^ *= 0.9906.* **d,** Experimentally measured power in a stationary film (film 1) as a function of x, y, and z distance between the center of two ME devices at a z-height of 1 cm below the coil, n = 3 measurements of the same samples for each data point, error bars represent standard deviation. The power decreases as the ME films come within 10 mm of each other along the x and y axes. **e,** Cross section of a COMSOL simulation showing a lower magnitude of the magnetic field in two films close to each other than two far apart films.

Unlike many wireless network technologies, we found that the WPT efficiency of a magnetoelectric network *increases* as we add more receivers. This feature results from the fact that the magnetic field is generated in a volume around the transmitter, and the devices place a negligible load on the transmitter. Experimentally, we found that the system efficiency increased from 0.22% WPT efficiency with one implant to 1.3% for 6 implants (Fig. 2a). We characterized PTE to a single device as a function of magnetic field strength (Fig. S1). We found a linear relationship between axial magnetic field strength and received ME film voltage and, therefore, a quadratic relationship between axial field strength and received ME power. By simulating the magnetic field produced by the transmitter in COMSOL, we calculated that the PTE to a single ME film is greater than 0.1% within a volume of approximately 60 cm^3^ and greater than 0.2% within a volume of approximately 34 cm^3^ (Fig. 2b). These values match our experimental PTE measurements (Fig. 2a) and field strength measurements (Fig. S2).

We also found that 7 × 3 mm ME films could be placed within 1 cm of each other without affecting the amount of power each film received from the transmitter. Since the Metglas layers of the ME film have high relative permeability (μ_r_ = 45 000), there is a limit to how close two ME films can be placed before the magnetic field that passes through each film is altered by the presence of a nearby film. We experimentally measured how close two films could be before they could no longer be treated independently by placing two vertically oriented films 1 cm below a magnetic field transmitter and measuring the output power of one film as we brought another film closer along the x, y, or z axes (Fig. 2d). As expected when two films approach each other along the x and y axes, we observe a drop in received power when the centers of the films are less than roughly 1 cm apart (Fig. 2d). At distances greater than 5 mm apart, there is less than a 5% drop in efficiency. This suggests that at centimeter scale distances, the films can be treated independently, and the received power can be estimated by the strength of the magnetic field at the position of the receiving film. We verified this result with a COMSOL simulation, showing that as the films move within roughly 1 cm along the y-axis, the magnetic field strength at the center of the film is weaker than it is when the films are more than 1 cm apart (Fig. 2e). Along the z-axis; however, the films benefit from concentrating the magnetic field along the long axes of the films (Fig. 2d, third panel). Indeed, when the film centers are 7.5 mm apart (edges in contact with each other) along the z-axis, the power is increased by nearly 40 %.

Understanding that the power transferred to a network of films separated by 1 cm or more can be estimated by the field strength at the film position, we simulated a network of up to 50 films and found an estimated PTE of up to 7.1 % with an inductance change in the transmitter of only 1.4 %. For these simulations, we modeled and estimated the system PTE and studied how each additional device affected the PTE of an array of 7.5 mm × 3 mm ME films below the transmit coil. We approximated the loading of the transmitter by the devices by calculating the change in inductance of the transmitter with each added film. For these simulations, we arranged the network of films in a grid spaced 1 cm apart in the x and y axes and at a z-distance of 1 cm below the coil. This allowed us to fit 25 films above the coil in a single layer. We found that this network of 25 films causes a change of inductance in the TX coil of less than 1% (Fig. 2c). We calculated the PTE of the network by extracting the magnetic field vector at each location and calculating the power based on the axial magnetic field strength (Fig. S1). To account for the direction of the magnetic field at each location, we experimentally found the relationship between received power and the angle of the incident magnetic field (Fig. S3) and used the fit equation to scale the received power at each location. We found A theoretical efficiency of up to 5.2 % could be achieved with a network of 25 ME films at a distance of 1 cm below the TX. To increase the network size further to 50 devices, we added a second layer of 25 ME films below the first at a distance of 2 cm from the TX coil. This configuration of 50 films in two layers produced an estimated system efficiency of 7.1 % and an inductance change of only 1.4 % in the TX coil (Fig. 2c).

## A network of spinal cord stimulators in a large animal model

We were able to independently activate a network of four ME-powered implantable spinal cord stimulators, resulting in the recruitment of four distinct muscle groups in the pig hindlimb. Spinal cord stimulation (SCS) is extensively used clinically for pain treatment ^16^ and is being investigated to restore movement for patients with paralysis due to spinal cord injury (SCI) ^17–19^. Recent findings demonstrate that SCS can differentially activate peripheral muscles ^20,21^, based on the anatomical arrangements of targeted motor pools within the cervical or lumbosacral enlargements. An optimized stimulation configuration and sequence can direct electric fields toward specific subsets of dorsal root entry zones, thereby modulating selected ensembles of motor neuron pools. This approach aids in enhancing specific motor functions with greater precision and objectivity, aligning with the priorities of targeted neurorehabilitation ^17,19,22^. However, achieving selective activation of the motor pools remains a significant challenge with current SCS techniques, necessitating further research to optimize stimulation parameters and enhance selectivity. Modern systems have up to 32 contacts in paddle arrangements and up to 16 contacts in linear arrangements ^23^, all tethered to a single battery-powered IPG. This architecture makes further increases in contact numbers challenging and limits the placement of the IPG in the body. One solution for both of these problems is a distributed system of smaller, battery-free devices with fewer contacts each that can be placed exactly where needed.

**Fig. 3 |:**
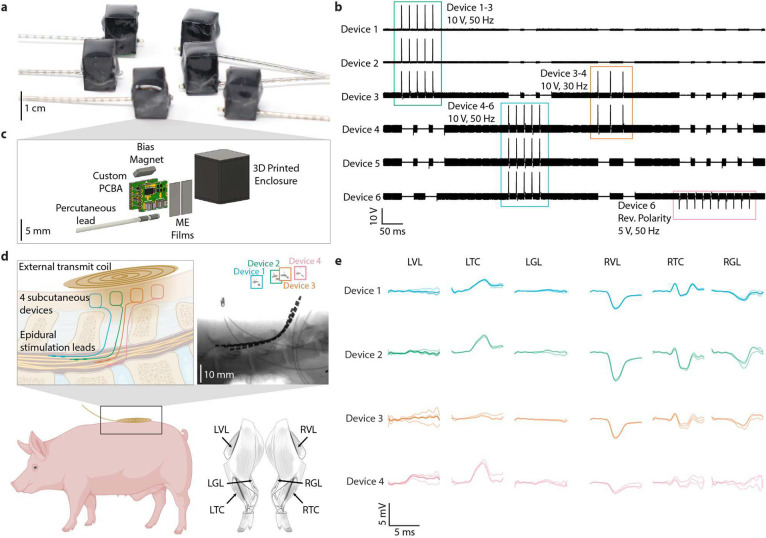
A wireless network for epidural spinal cord stimulation. **a,** A network of 6 single-channel IPGs connected to stimulation leads **b,** An example sequence showing the output from 6 devices and on-the-fly reconfigurability of the entire network with a single transmitter. **c,** A detailed view showing the internal components of the ME-powered IPG. **d,** (inset, left) Schematic of surgical setup with 4 ME-powered devices placed subcutaneously and leads extending into the spinal epidural space. (inset, right) X-ray image showing the placement of the four leads used for the first study. (bottom, right) Locations of EMG measurements in the pig leg muscles. **e,** Recorded EMG responses in six muscles in response to stimulation with four wireless devices activated separately. Each signal is plotted with three trials and the mean from those three trials.

In our system, each device consists of a wirelessly powered, battery-free IPG approximately 1 cm by 1 cm by 1 cm in size, capable of delivering digitally programmable voltage-controlled stimulation pulses up to an amplitude of 14.5 V. The IPG circuitry is as previously described in Woods et al. 2024 ^24^. The circuit consists of rectification, storage capacitance, a programmable output boost converter, and a microcontroller. We built six devices, each packaged with a 3D-printed enclosure and epoxy and connected to a single pair of contacts on a commercially available spinal cord stimulation lead (Fig. 3a,c). Like the earlier described devices, digital data is encoded from the transmitter by turning the powering magnetic field on and off for specified durations. We can program each of the 6 devices to a specified amplitude and trigger stimulation pulses on specific subsets of the network at various frequencies (Fig. 3b). We also developed a graphical user interface for ease of use in the operating room (Fig. S4).

**Fig. 4 |:**
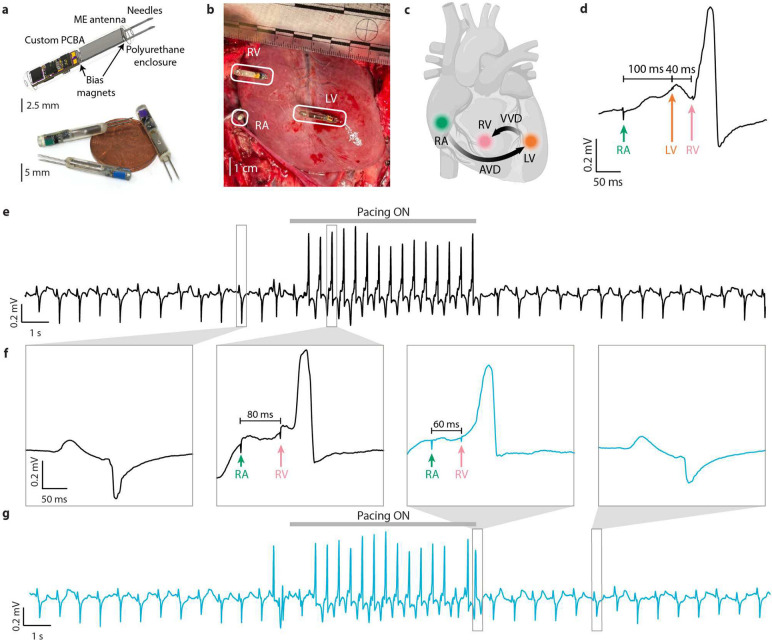
A wireless network for cardiac pacing. **a,** (top) pacing device detail (bottom) 3 pacing devices with a US penny for scale. **b,** 3 pacing devices placed on the right atrium (RA), right ventricle (RV), and left ventricle (LV). **c,** a schematic of the pacing locations and associated atrioventricular delay (AVD) and inter-ventricular delay (VVD). **d,** A trace from ECG 12-lead, lead II showing a paced beat with an AVD of 100 ms and a VVD of −40 ms. **e,** A trace from 12-lead ECG lead II showing a period of pacing with 80 ms AVD. **f,** (left) an unpaced beat before 80 ms AVD pacing is turned on (center-left) a zoom-in of a paced beat with 80 ms AVD (center-right) a zoom-in of a paced beat with 60 ms AVD (right) an unpaced beat after 60 ms AVD pacing is turned off. **g,** a trace from 12-lead ECG lead II showing a period of pacing with 60 ms AVD.

We used a system of four devices to apply stimulation epidurally over the spinal cord in a porcine model. We performed a laminectomy of the L6 vertebra, and the leads were implanted into the dorsal epidural space between L4 and L5 vertebrae and sutured into place. This location was specifically chosen as it corresponds to the lumbar enlargement of the spinal cord ^25,26^, which encompasses motor pools that project to the hindlimb muscles. The IPGs, which were attached to the leads before implantation, were then placed in a subcutaneous pocket and were powered and programmed from the surface of the skin with a single external transmitter (Fig. 3d) while a separate system simultaneously recorded electromyography (EMG) to measure evoked responses from the left and right vastus lateralis (LVL and RVL), cranial tibial (LTC and RTC), and gastrocnemius lateralis (LGL and RGL) muscles. With two devices in an initial surgery, we could observe selective proximal (i.e., VL) and distal (i.e., TC and GL) muscle activation when the devices were activated sequentially, with the activity superimposed when activated synchronously, and a titrated muscle response in response to increasing stimulation amplitudes (Fig. S5). With all four devices stimulating sequentially at 13.75 V while powered by a single transmitter, we observed distinct muscle activity with each device (Fig. 4e). We processed the data using wavelet decomposition and principal component analysis and can observe four distinct clusters corresponding to the evoked response from each device (Fig. S6).

## A network of wirelessly powered pacemakers in a large animal model

The reliable power and data transfer of ME-powered bioelectronic networks allowed us to coordinate stimulation patterns from multiple cardiac pacing nodes in a porcine heart failure model despite the centimeter-scale movement experienced by devices placed on a beating heart. Multi-chamber cardiac resynchronization therapy (CRT) relies on pacing from multiple locations with precise timing to resynchronize the heart’s rhythm in patients with congestive heart failure (CHF) ^27^. However, increasing the number of pacing locations is limited by the number of leads that can be routed into the heart ^28^ or, in the case of recent wire-less pacemaker technologies, by the size of battery-powered leadless devices ^29^. Our pacing system consists of a network of up to 3 wireless and battery-free ME devices (Fig 4a), each placed in a different chamber of the heart. These devices are powered by an external two-lobe transmitter coil placed at a nominal distance of 4 cm from the surface of the heart (Fig. S7) and controlled by a user interface on a PC (Fig. S8). In this example, we placed a device each on the right atrium (RA), right ventricle (RV), and left ventricle (LV)(Fig. 4b) of a porcine model (n = 3) with induced heart failure. We then applied pacing with a programmed atrioventricular delay (AVD) and inter-ventricular delay (VVD) to provide biventricular therapy (Fig. 4c). An example tracing of a paced heartbeat with an AVD of 100 ms and a VVD of −40 ms (LV before RV) is shown in Figure 4d.

With this system, we could coordinate the relative timing of three independently addressable pacing nodes on the surface of a beating porcine heart, a feature necessary for optimal clinical outcomes. The system achieved consistent biventricular pacing with clearly visible pacing artifacts in the electrocardiogram. When pacing was activated, we observed an increase in heart rate from the native rate of 78 beats per minute (BPM) to the paced rate of 140 BPM, and when pacing was deactivated, we observed a return to the native rate (Fig. 4e,g). Zooming in on paced heartbeats, we can see a noticeably different shape of the QRS complex from the native (Fig. 4f) and visible pacing artifacts with programmed delays of 80 ms AVD and 60 ms AVD. This reflects and validates various proportions of paced versus native myocardial activation observed with different timing intervals. The pacing parameters and resulting QRS complex widths are summarized in Supplementary Table 1. This demonstrates the ability of ME WPT to power and control a network of pacing devices from a large distance in the dynamic environment of the beating porcine heart.

## Discussion

This work shows the potential of ME power to enable scalable networks of wireless, battery-free devices for neurostimulation. Because magnetic fields at this frequency experience low losses in biological tissue at a frequency of 220 kHz, ME devices can be powered with mT-strength magnetic fields that extend over centimeters within the safety limits for biological tissue. This broad magnetic field can power many ME devices simultaneously with minimal transmitting coil loading, leading to system PTEs that increase with the network size. The simplicity and robustness achievable with a wireless network implemented using ME power could support many new applications for implantable networks.

The two large animal demonstrations shown here highlight the ability of ME-powered bioelectronic networks to reach stimulation voltages over 10 V and operate reliably under centimeter scale displacements, which are key features for any technology intended for clinical translation. The challenge of delivering mW-level powers wirelessly to many devices simultaneously with centimeter-scale misalignment tolerance may have prevented demonstrations of large bioelectronic networks using other wireless power technologies. These types of networks may prove useful for increasing the number of stimulation contacts and placing them exactly where they are needed without the concern of routing multiple wires to a single large IPG. The miniaturization afforded by making these implants battery-free also facilitates minimally invasive surgical delivery, perhaps through the blood vessels and into both chambers of the heart in the case of cardiac pacing.

Some limitations of ME-powered bioelectronic networks include a relatively low PTE compared to larger centimeter-sized wirelessly powered implants. Battery-powered transmitters can support many hours of ME-powered device operation ^15^, but the size of the battery in the wearable could likely be reduced if one increases the size of the implant to several centimeters and uses more efficient inductive WPT ^30^. This fact can be offset by the fact that ME power scales with receiver area rather than receiver area squared like inductive coupling and that the safety limits for 220 kHz fields are orders of magnitude higher than safety limits at 1 GHz ^31^ as used in RF power transfer ^1^. Indeed if miniaturization, networking, or high powers are not needed, ME-powered devices may not be the best wireless power solution for implantable bioelectronics ^11^. Future work is also needed to facilitate multichannel uplink from the wireless network. While near-zero-power backscatter uplink is possible from a single ME-powered device ^32^, receiving data from many ME-powered devices in a network may require an alternative uplink communication method or advances in ME backscatter communication protocols to support multiplexed uplink.

Distributed bioelectronic networks supported by the ME-power and data strategy shown here have properties that could overcome limitations associated with current bioelectronic architectures. Distributed wireless systems can be smaller, placed precisely where needed for each patient, and reduce the need for complex, high-density feed-throughs in device packages. Indeed, the two in vivo demonstrations shown here support bioelectronic applications with different requirements in terms of power, distribution of nodes, and tolerance for device movement, yet we could support them both with relatively minor changes to the underlying elements of the network. Combining this versatile bioelectronic platform with sensing and data uplink, future systems promise to become intelligent networks of implanted and wearable devices that coordinate their activity throughout the body like a mesh network to monitor, modulate, and improve human health.

## Methods

### Fabrication of ME Films

We manufactured ME films by first epoxying (M-Bond 43-B) PZT (Piezo Systems PZT 5H PSI-5H4E) and Metglas (2605SA1, Metglas Inc) into a three-layer laminate with Metglas on both sides of the PZT. Next, we cut the laminate with a femtosecond laser (One Five Origami XP, NKT Photonics) into 7.5 by 3 mm individual films. We used a symmetric pattern to allow us to cut through from one side, flip the laminate, and cut from the other. Once cut, we tested films at resonance in a 220 kHz alternating magnetic field and only kept those with an open-circuit peak-to-peak voltage of more than 25 V. Approximately 80% of cut films met this criterion.

### Blinking Network Demonstration

We designed a custom printed circuit board assembly (PCBA) to receive energy from the ME films and digitally control LEDs. It consists of a Schottky diode bridge rectifier, supervisor circuit, two 22 uF storage capacitors, communication resistors, a 2 V low-dropout regulator, a blue LED (Kingbright APHM1608VRBXF) and a microcontroller (NXP KL02). The supervisor circuit is used to prevent large inrush current from preventing the device from booting. It is implemented with a voltage supervisor (Analog Devices MAX16072RS17) connected to the gate of an N-channel MOSFET, which connects the microcontroller ground to true ground once a capacitor is charged. The blue LED is attached in parallel with a 2 kiloohm resistor and in series with another 22 μF capacitor and driven by two high-current drive general-purpose input output pins from the microcontroller with opposite polarity during a pulse. This allows the application of a 1 ms duration, 4 V amplitude pulse across the LED from the 2 V supply rail ^33^. A digital ID is encoded in each device’s firmware to allow individual addressability. Downlink data encoded in the transmitter magnetic field is decoded by the microcontroller using a resistive divider on the rectified voltage input into the onboard comparator.

A 7.5 mm by 3 mm magnetoelectric film is connected to the PCBA with Teflon-coated copper wire (Cooner Wire CZ1187) and conductive epoxy. It is then folded over to be parallel with the PCB. The film is biased to optimal resonance using a 1 mm × 3 mm × 0.25 mm neodymium magnet (Supermagnetman Rect 0010–10), which is cut in half, insulated with Kapton tape, and placed at each end of the film. The film is then protected by cutting shrink tubing in half, wrapping it around the film, and supergluing it to the edges of the PCB.

For the transmitter, we used a 9.5 cm outer diameter, 5 cm inner diameter, single layer, planar, spiral coil optimized to produce a 5 cm diameter circular area of uniform magnetic field strength to transmit power to the films. The coil is tuned to series LC resonance to match the 220 kHz resonant frequency of the films. The coil is driven at 220 kHz by a custom H-bridge driver. For this experiment, we used a transmit power of 10 W, corresponding to a measured magnetic field strength of 2 mT at the coil’s surface. The logic is controlled with an Analog Discovery Pro function generator. The devices are programmed sequentially on or off, and blinking is triggered at a rate of 100 Hz with a 1 ms pulse width. Therefore each ‘blink’ in the video or photo frames actually consists of many 1 ms flashes of the light at 100 Hz, which is not visible as flashing but rather consistent light.

### PTE Measurements

To characterize the efficiency of simultaneous power transfer to a network of independent ME films, we used the same TX coil as in the blinking network demonstration. We placed six films below the TX coil with the bottom edge of each film 1 cm from the coil’s surface and measured output AC voltage across the optimal load resistance of 1.33 kiloohms for all six films simultaneously, as we removed one film at a time from below the coil. The TX coil power output remained at a constant 1 W without changing the TX circuit’s capacitance to maintain a resonance condition. At this power output, the maximum magnetic field strength at the surface of the TX coil is approximately 0.64 mT RMS, much lower than the safety limit of 8 mT RMS ^15^. We found that the PTE from the coil to the films increases linearly with each additional film that is added: a single film receives 2.2 mW at this distance, a PTE of 0.22%, but six films together each receive 2.2 mW - 13.2 mW in total - corresponding to a PTE for the full network of approximately 1.3%. This shows the potential for efficient simultaneous power transfer to networks of ME-powered implantable devices.

To calculate the power transfer efficiency of both individual ME films and networks of ME films, we compared the power in the transmit coil to the combined received power by each ME film in the network. We measured power in the transmit coil by measuring the peak AC current in the TX coil ($I_{pk}$) and the resistance of the TX coil at the resonant frequency (R=0.26Ω). We then calculate root mean square (RMS) alternating current (AC) power as:

$$P_{RMS,TX}={\left(\frac{I_{pk}}{\sqrt{2}}\right)}^{2}\;*\;R$$

We measured the power in the ME films by measuring the peak AC voltage $\left(V_{pk}\right)$ across an optimal load resistance of $R_{L}=1.33$ kiloohms. We then calculate RMS AC power as:

$$P_{RMS,FILM}={\left(\frac{V_{pk}}{\sqrt{2}}\right)}^{2}/R_{L}$$

We then calculate the network power transfer efficiency (PTE) as the sum of film power divided by the transmit power:

$$PTE\;(\%)=\frac{\Sigma \;P_{RMS,FILM}}{P_{RMS,TX}}\;*\;100$$

### Network scalability simulations

We measured the magnetic field strength below the coil at various locations using an alternating magnetic field probe (AMF Lifesystems) and compared that to the ME film voltage. A linear relationship exists between the ME film received voltage and the axial magnetic field (Fig. S2a). This linear relationship with voltage corresponds to a quadratic relationship between the PTE and the axial component of the magnetic field (Fig. S2b).

We simulated the magnetic field produced by the transmit coil in COMSOL Multiphysics. We duplicated the geometry of the 9.5 cm outer diameter, 5 cm inner diameter, single layer, planar spiral coil used for testing. We placed this coil in a 10 cm radius sphere. We used the magnetic field module to apply 2.85 amps of current in the coil and plotted the output magnetic field for visualization. We also extracted the magnetic field along 3 lines: two parallel to the x-axis at z = 1 cm and z = 2 cm, and one along the z-axis. The simulation closely matched the experimentally measured values of the axial magnetic field (Fig. S4), so we used these values with the efficiency at different axial magnetic field strengths to plot efficiency over volumes. To calculate the transmitter inductance as a function of network size, we used the same model but added ME laminates in a grid above the coil, as described in the main text. We then measured the coil inductance using the *mf.LCoil_2* expression in COMSOL.

### Epidural Spinal Cord Stimulators

We used the same PCBs, components, and general assembly procedure for the epidural spinal cord stimulation devices as described in Woods et al. 2023 ^24^. The circuit consists of power rectification and storage, an adjustable output boost converter to generate the stimulation rails, a microcontroller for digital stimulation control, and an output switch to modulate stimulation output. We assembled two PCB panels by hand in the laboratory and joined them together using castellated vias on the edges of the PCBs and 3 mm lengths of uninsulated wire. After joining, we tested the devices’ functionality and attached ME films using vertically soldered 0 Ohm resistors and conductive epoxy. We fixed a 1 mm by 2 mm neodymium bias magnet on the PCB assembly to bias the ME films at their optimal length resonant mode. We designed the firmware on the devices to accommodate individual addressability using the state machine shown in Fig. 4b. For this work, we connected the two contacts on the end of percutaneous spinal cord stimulation leads (Infinion 16-contact percutaneous lead; Boston Scientific, USA) to the stimulation contacts on the PCBs using conductive epoxy. We then enclosed the devices for temporary implantation using a 3D-printed box (Projet MJP) and a two-part epoxy (Hardman Double/Bubble 210–0100) and tested the devices for full operation.

To show the programmability trace in Fig. 1c, we placed fabricated devices on a Teflon block 1 cm above a transmit coil. We used a two-layer pancake coil with an outer diameter of 10 cm and an inner diameter of 4 cm to power the devices. The transmit coil had an output power of 7 W for this testing. This high transmit power was required to overcome the relatively large inrush current of the off-the-shelf components used in the circuitry, which could be remedied in the future with more efficient ASICs. We measured the output of all six devices simultaneously using two four-channel oscilloscopes with a synchronization input from the transmitter. The transmitter was programmed to send the desired sequence of commands to all six devices while they were simultaneously powered and programmed by the transmitter. For the in-vivo testing, we used the same transmit coil and output power with a user interface for real-time control of the stimulation parameters during the experiment.

### In Vivo Acute Spinal Cord Stimulation Experiments

The animal procedures were conducted in accordance with the rules of the Institutional Animal Care and Use Committee (IACUC) of Houston Methodist Research Institute (Houston, TX) (STUDY ID: IS00006754). The housing, feeding, and general care of the animals were conducted in accordance with the guidelines set forth by the US Department of Agriculture and the Association for Assessment and Accreditation of Laboratory Animal Care (AAALAC). Throughout the course of the experiment, a veterinary professional with expertise in doing research on porcine animals was present. The protocol generally followed previous methods for mapping spinal circuitries ^26^.

Two female Yucatan minipigs (experiment 1, experiment 2), both aged 3 months, weighing 20.6 and 21 kg, respectively, were pre-anesthetized with an intramuscular injection of midazolam (0.3 mg/kg), ketamine (20 mg/kg), and hydromorphone (0.15 mg/kg), endotracheally intubated, ventilated, and maintained with isoflurane (2–4%) in the prone position. The temperature was monitored via a rectal thermometer and regulated with Bair Hugger blankets and a circulating warm water blanket.

A laminectomy of the sixth lumbar vertebra was performed, then two or four (experiment 1, experiment 2) Infinion 16-contact percutaneous leads (Boston Scientific, USA) were implanted into the epidural space. C-arm fluoroscopy was then used to confirm the correct lead positioning and the leads were sutured into place. The IPGs for the leads were placed into a subdermal pocket. Gas anesthesia was changed to a total intravenous anesthesia (TIVA) protocol prior to starting the electrophysiological testing. The TIVA protocol included dexmedetomidine (2 μg/kg/hr), ketamine (5 mg/kg/hr), and propofol (8 mg/kg/hr). Throughout the experiment, TIVA was adjusted to guarantee that a sufficient amount of anesthetic was administered while limiting the effects on stimulation and recording.

Subcutaneous needle electrodes (length: 20 mm; Rhythmlink Columbia, SC, USA) were used to record electromyography (EMG). The electrodes were positioned longitudinally over the left and right vastus lateralis (VL), semitendinosus (SEM), tibialis cranialis (TC), and gastrocnemius caput lateralis (GC). Electrode recordings were made using a PowerLab data acquisition system (ADInstruments) and amplified using a differential amplifier Octal Bio Amp (ADInstruments, Australia; common-mode rejection ratio: > 60 dB, gain: 100, range: ± 200 μV, resolution: 100 nV). The electrode signals were sampled at 10,000 Hz and recorded using LabChart ADInstruments (version 8.1.24). A subdermal needle electrode was placed over the right calcaneal tuber, which served as the ground electrode

We extracted an activity window after the trigger signal on all EMG channels and bandpass-filtered the data between 15 and 800 Hz. For all EMG signals presented in this work, we plotted three trials and the mean from those three trials. For recruitment curves, we plotted the signal along with the mean and standard deviation of the peak amplitude for each stimulation level. To visualize the difference in recruitment for different channels in Fig. S6, we used wavelet decomposition with a level 3, order 8 Daubechies wavelet, then plotted data using the first two principal components across all channels and the first 5 wavelet coefficients.

### Cardiac Pacing Devices

For the cardiac pacing devices, the circuit was the same as described in the ‘Blinking Network Demonstration’ section without the LED or 2 kΩ resistor. 3 devices were built and color-coded according to their intended placement on the RA, RV, or LV. A 7.5 mm × 2.5 mm ME laminate was attached to the PCB on the end in the same plane as the PCB using Teflon-coated copper wire (Cooner Wire CZ1187) and conductive epoxy. The film is biased using a 1 mm × 3 mm × 0.25 mm neodymium magnet (Supermagnetman Rect 0010–10), which is cut in half, insulated with Kapton tape, and placed at each end of the film. Stainless steel needle electrodes are soldered to the PCB (Technomed TE/S43–438) so the tips extend 5 mm past the film’s end. A tube is made of medical grade polyurethane (Dymax 1128A-M) by coating a 2.76 mm diameter polytetrafluoroethylene (PTFE) rod (McMaster-Carr 84935K86). The entire device is then inserted into the polyurethane tube, and the ends are sealed with the same polyurethane epoxy.

A 3-bit communication packet sent to all devices by modulating the on-off timing of the transmit coil encodes 8 separate commands, which allow individual pacing from each of the 3 sites at 4 V amplitude with a pulse width of 0.5 or 1.0 ms. The remaining two commands are used to enable pacing from the LV and RV simultaneously with a pulse width of 0.5 or 1.0 ms. With the 3 data bits and a start and stop bit, each downlink command only requires 1.25 ms to send, allowing externally programmable delays between devices as low as 2 ms. To ensure the reliability of data transfer to the devices while still sending information quickly, all 3 bits must have been received within 1.25 ms or the device ignores the downlink command and does nothing.

### In Vivo Acute Cardiac Pacing Experiments

The animal procedures were conducted in accordance with the rules of the Institutional Animal Care and Use Committee (IACUC) of Texas Heart Institute (Houston, TX) (STUDY ID: 2024–02). We validated the system in a porcine model (n = 3) with induced heart failure. For inducing heart failure, a dual chamber pacemaker (Assurity MRI(TM) 2272, Abbott, MN) was connected to a lead on the RV apex (Tendril(TM) STS 2088TC) and programmed to continuously pace the right ventricle for a period of 4 weeks or until the left ventricle ejection fraction (LVEF) dropped below 35% indicative of heart failure. RV apical pacing is an accepted model for inducing CHF in large animals. In the first week of implant, the heart was paced at a nominal rate of 120 BPM. Diagnostic echocardiograms to assess LVEF were obtained weekly to assess the progress of the heart failure. The pacing rate was gradually increased by 10BPM every consecutive week if the LVEF had not diminished to the target threshold. After the LVEF dropped below 35%, the animal was prepared for a non-survival study. This heart-failure model causes the animal to be more likely to enter fibrillation, which resulted in the early termination of two of the three studies.

Animals were sedated via an intramuscular injection of Telazol 2–6mg/kg and Atropine Sulfate 0.02–0.05 mg/kg. Isoflurane was administered via a facemask to induce general anesthesia for surgical interventions. The pigs were intubated and appropriately prepped for surgery. IV catheters were established, and the animals were transported to the OR. Naxcel (Ceftiofur) 3–5 mg/kg was administered intramuscularly. Appropriate preoperative analgesics such as Buprenorphine HCL 0.005–0.1 mg/kg and Flunixin Meglumine 1.1–2.2 mg/kg IM, IV or SQ. (or equivalent to either) were administered. For this specific study, the anesthetic of choice is isoflurane (0.5–5.0%) and/or propofol (10–20 mg/kg/hr IV). The animal was placed on the table in the dorsal position. The chest was aseptically prepped with betadine. A midline incision was performed from the sternal notch to the xiphoid process using electrocautery. Once all of the soft tissues were divided, the sternum was opened using a sternal saw, followed by an oscillating saw for the manubrium. The sternum was then retracted using a self-retaining retractor made out of PVC. With the heart on sight, the thymus was resected, and the pericardium was opened with a T incision. Silks were used to create the pericardial cradle and stabilize the heart.

After performing a median sternotomy, pacing nodes were implanted on the right atrium, right ventricle, and left ventricle to provide biventricular pacing in an open-chest model. In the first animal, each node was individually addressed and tested; confirmation of pacing was assessed by investigating the 12-lead ECG. Confirmation of pacing from each individual node was established. However, while testing the device on the right atrium, animal 1 developed ventricular fibrillation and could not be resuscitated.

In animal 2, each of the nodes was successfully validated. After inducing heart failure, the QRS width increased from 60 ± 0 ms to 64 ± 0 ms. Pacing was performed at a fixed output voltage of 4V at a pulse width of 0.5ms. A two-lobed magnetic field transmitter was placed above the chest to cover the entire heart. Pacing was performed with i) atrioventricular delay (AVD) of 100 ms with inter-ventricular delay (VVD) of −40 ms (VVD < 0 implies LV was paced before RV), resulting in a QRS width of 71.67 ± 2.89 ms; ii) AVD of 100 ms and VVD of −20ms resulting in QRS width of 72 ± 4 ms; iii) AVD of 100 ms and VVD of 0 ms resulting in a QRS width of 81 ± 5.03 ms; iv) AVD of 80 ms and VVD of 20ms resulting in a QRS width of 74 ± 6.93ms; and v) AVD of 60ms and VVD of 40ms resulting in a QRS width of 114.67 ± 8.33 ms The animal developed ventricular fibrillation when being paced with an inter-ventricular delay of 40ms and could not be resuscitated. 12-lead ECG and pressure data were captured for each pacing event and analyzed. Pacing artifacts clearly visible on the 12-lead ECG indicated that pacing was being performed with the programmed delays.

In animal 3, induction of HF increased QRS width from 50.67 ± 1.15 ms to 82.67 ± 4.62 ms. While testing the nodes individually, it was noted that one of the pacing nodes was not performing reliably. A decision was made to continue the study with two nodes and to pace only from the right atrium and the right ventricle with differently programmed atrioventricular delay. Pacing was performed at a constant voltage of 4V and pulse width of 0.5ms. A single transmitter was used to power and program both nodes. Pacing was performed with i) AVD of 60 ms resulting in a QRS width of 65 ± 10.44 ms; ii) AVD of 80 ms resulting in a QRS width of 62 ± 0 ms; and iii) AVD of 100 ms resulting in a QRS width of 69.33 ± 6.11 ms.

## Supplementary Material

Supplement 1

## Data Availability

The main data supporting the results of this study are available within the paper and its Supplementary Information.
