# Supplementary material for: Scalable networks of wireless bioelectronics using magnetoelectrics
Source: Res Sq. 2024 Sep 24:rs.3.rs-5005441. Preprint. [Version 1] doi: 10.21203/rs.3.rs-5005441/v1 (PMC11469518; doi:10.21203/rs.3.rs-5005441/v1)
Supplement: Supplement 1 [file NIHPPRS5005441V1-supplement-1.pdf]

Supplementary Materials

		QRS Width (ms)		
		**Animal 01**	**Animal 02**	**Animal 03**
HF induction				
Baseline		58 ± 2	60 ± 0	50.67 ± 1.15
Baseline - post HF		64 ± 2	64 ± 0	82.67 ± 4.62
Therapy				
AVD	VVD			
100	-40		71.67 ± 2.89	
100	-20		72 ± 4	
100	0		81 ± 5.03	
80	20		74 ± 6.93	
60	40		114.67 ± 8.33	
60	-			65 ± 10.44
80	-			62 ± 0
100	-			69.33 ± 6.11

Supplementary Table 1 | QRS width as a function of AV and VV delays
Average and standard deviation of QRS width as measured from 3 representative captured heartbeats. The baseline width is narrower than the width after heart failure (HF) induction. Pacing with different atrio-ventricular delays (AVD) and inter-ventricular delays (VVD) results in different QRS widths than baseline. In general, shorter QRS complex widths imply more homogeneous cardiac activation and efficacy of CRT.

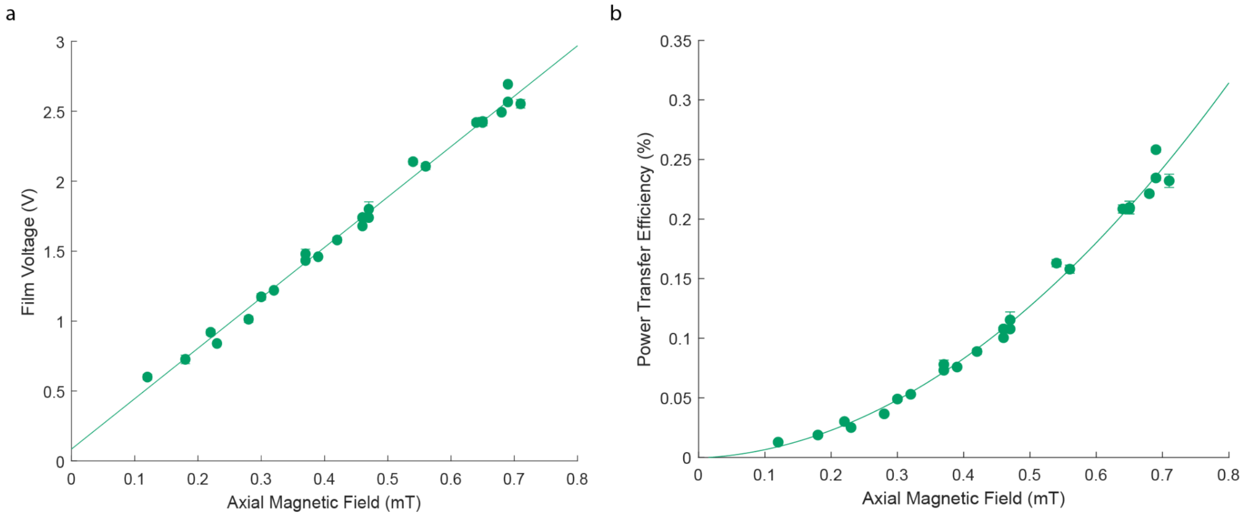

Supplementary Figure 1 | Relationship between received power and axial magnetic field strength.

a, Measured results show a linear relationship between ME film voltage and axial magnetic field strength. Fit line: $3.60x + 0.09$, $R^2 = 0.9932$, n = 3 measurements of the same samples for each data point, error bars represent standard deviation. **b,** Quadratic relationship between axial magnetic field and PTE. $0.46x^2 + 0.02x$, $R^2 = 0.9910$, n = 3 measurements of the same samples for each data point, error bars represent standard deviation.

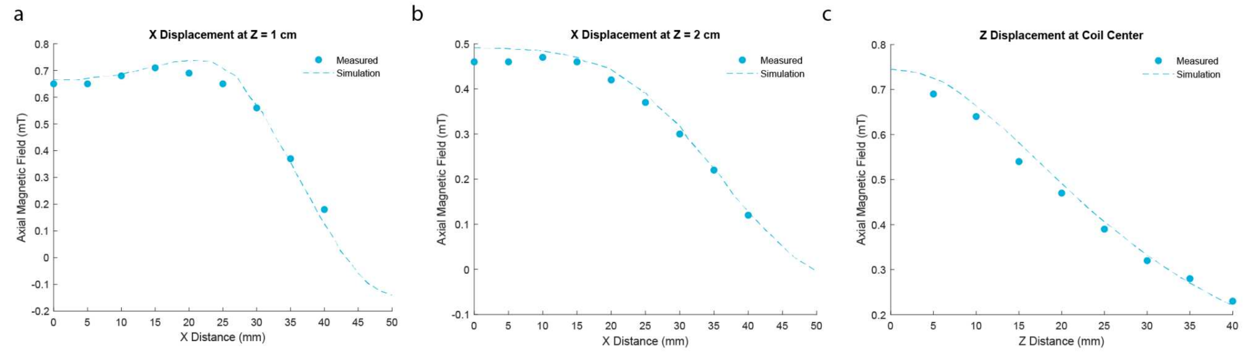

Supplementary Figure 2 | COMSOL model vs experimental measurements.
a, Measured axial magnetic field compared to the calculated axial magnetic field using COMSOL at a z-distance of 1 cm along x displacement from the center of the coil, x = 0, to x = 40 mm away. **b,** Comparison with x displacement at a z-distance of 2 cm. **c,** Comparison with z displacement at the center of the coil.

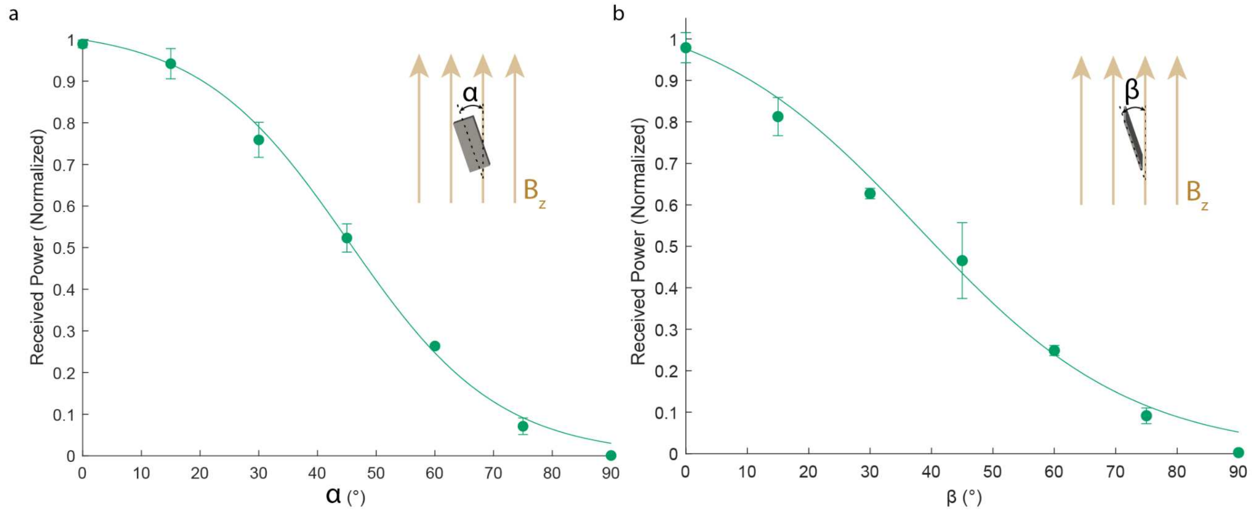

Supplementary Figure 3 | Relationship between received power and rotation angle.
a, Measured results (n = 3 measurements each point) show a sigmoidal relationship between ME film received power and angle relative to the magnetic field. Sigmoidal Fit: *1.03/(1 + exp(0.08 * (x - 45.34)),* $R^2 = 0.9977$, n = 3 measurements of the same samples for each data point, error bars represent standard deviation. **b,** Sigmoidal Fit: *1.09/(1 + exp(0.06 * (x - 38.03)),* $R^2 = 0.9878$, n = 3 measurements of the same samples for each data point, error bars represent standard deviation.

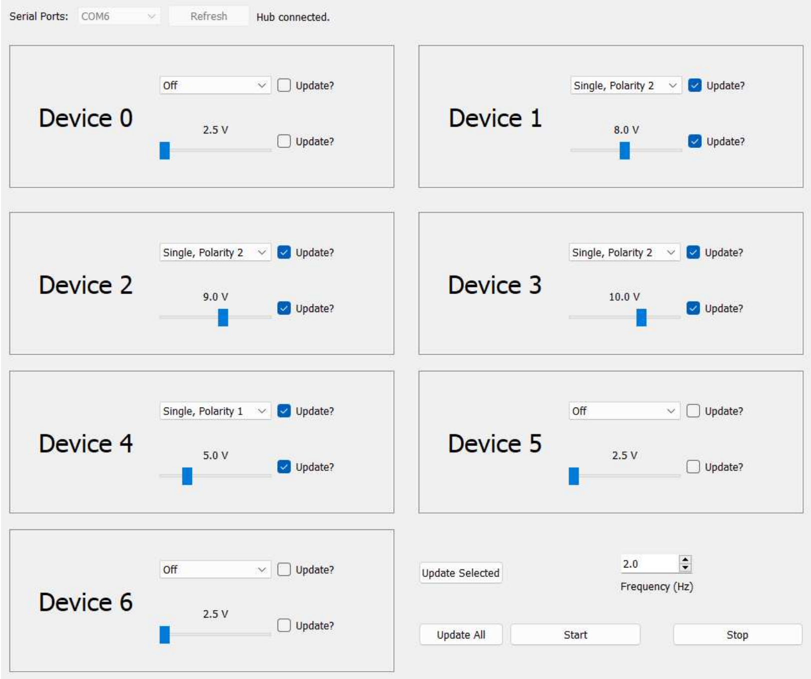

Supplementary Figure 4 | Graphical user interface for configuring spinal cord stimulation network.

The user interface allows users to turn up to six devices on and off and select the stimulation output polarity and amplitude.

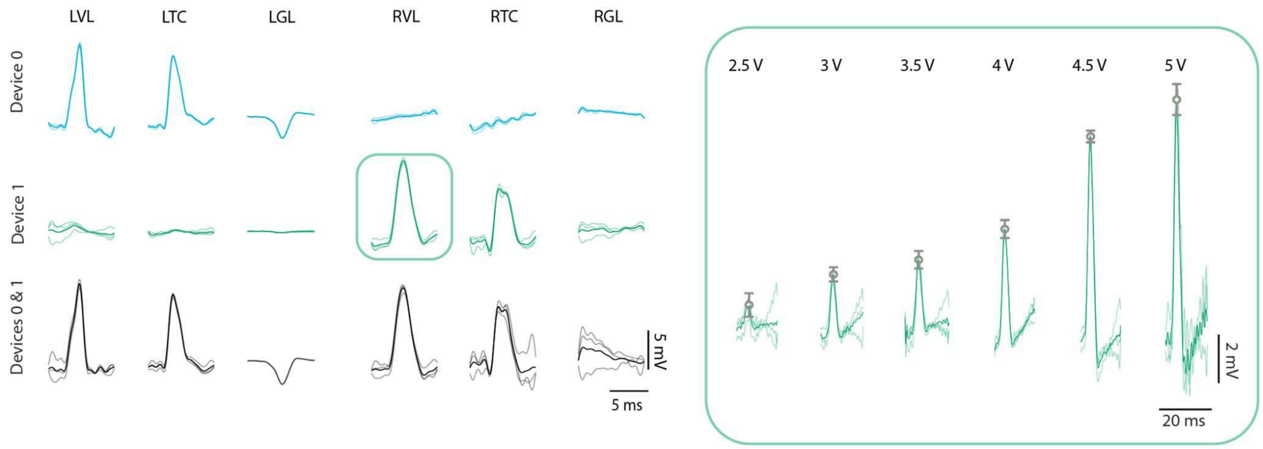

Supplementary Figure 5 | Programmable spinal cord stimulation with 2 devices. Recorded EMG responses in six muscles in response to stimulation with two wireless devices activated separately and synchronously. (inset) Increasing muscle movement in the RVL muscle in response to increasing stimulation amplitude from device 1.

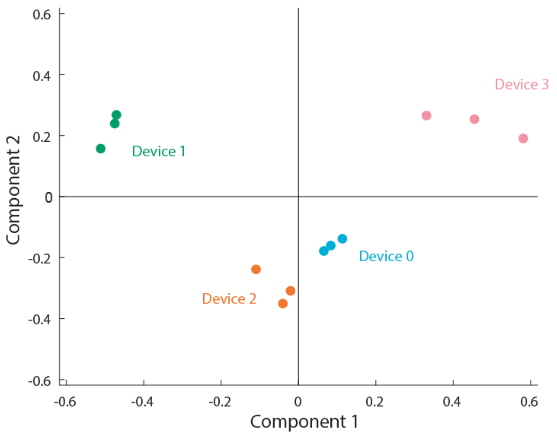

Supplementary Figure 6 | Separability of muscle responses to stimulation by 4 devices. Clustered responses along two principal components show the separability of muscle responses to stimulation by different devices.

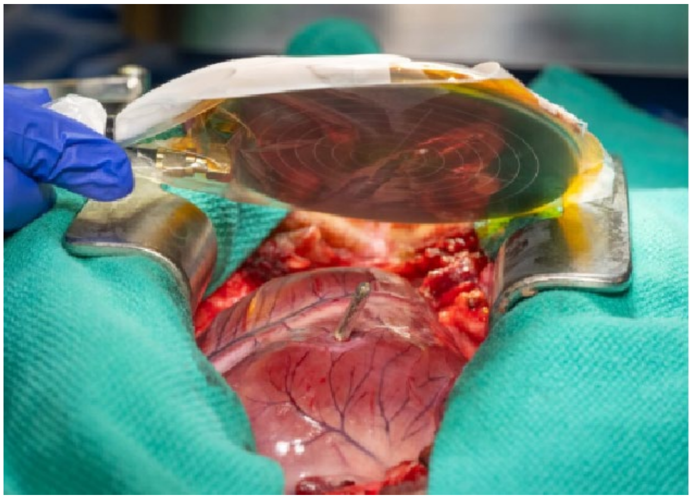

Supplementary Figure 7 | Image showing relative placement of a wireless device and transmitter on the surface of a pig heart.

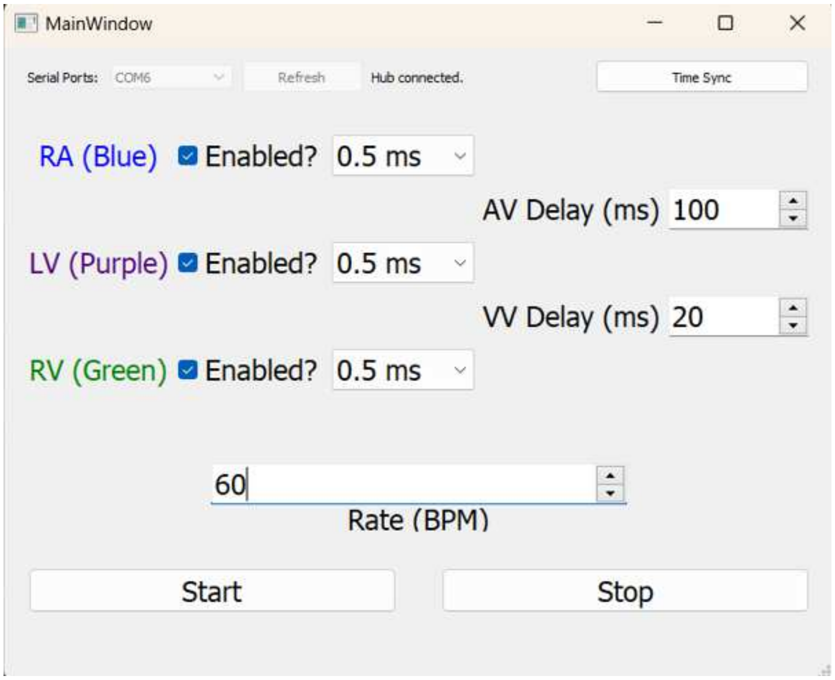

Supplementary Figure 8 | Graphical user interface for configuring cardiac pacing network. The user interface allows users to enable and configure the pulse width, AVD, VVD, and pacing rate for a network of 3 cardiac pacing devices.

Movie 1 | A network of 12 wirelessly powered and programmed ME devices.

The video shows a programmed sequence of blinking devices, all simultaneously powered and programmed by a single transmitter. The devices can be moved while still receiving power and data, as shown in the first segment by moving one of the devices. Arbitrary sequences can be programmed rapidly as shown in the second segment.

Supplementary Files

This is a list of supplementary files associated with this preprint. Click to download.

- SupplementaryMovie1.mp4
